# The Red Backgrounds of Wall Paintings from Isturgi and Cástulo (Jaen, Spain): A Multi-Technique Approach to Understanding and Improving Their State of Conservation

**DOI:** 10.3390/ma18071533

**Published:** 2025-03-28

**Authors:** A. I. Calero-Castillo, T. López-Martínez, M. Calero, M. J. Muñoz-Batista

**Affiliations:** 1 Department of Painting, Faculty of Fine Arts, University of Granada, 18074 Granada, Spain; tlopez@ugr.es; 2 Department of Chemical Engineering, University of Granada, 18074 Granada, Spain; mcaleroh@ugr.es

**Keywords:** red pigments, wallpainting, heritage, conservation, material characterization

## Abstract

This contribution presents a multidisciplinary approach that encompasses contextualization, photographic, and graphic documentation, as well as a comprehensive characterization scheme focusing on the morphological, chemical, structural, and electronic aspects of the red panels from two significant archeological sites: Cástulo and Isturgi. The red panels, which constitute the predominant component of the paintings, are indicative of their conservation state and were characterized using various techniques, including several microscopies tools, X-ray diffraction, X-ray photoelectron spectroscopy, and Fourier-transform infrared spectroscopy. The characterization scheme revealed significant structural differences in the paintings, with hematite present in the samples from Isturgi and both hematite- and lead-containing components being observed in those from Cástulo. The organic components are primarily associated with the use of Paraloid during the extraction of the paintings. Notable differences related to the encrustations of carbonate species are also observed, which are more prominent in the paintings from Cástulo. The results enable a discussion of their state of deterioration and the identification of future lines of action for their conservation.

## 1. Introduction

The conservation of Roman mural paintings presents a complex challenge, particularly due to the specific characteristics of these types of decorations. A critical factor is the wallpainting composition, encompassing both the pigments and the mortar used, along with the environmental conditions under which they were preserved, whether in archeological sites or in a museum setting. These aspects greatly influence the effectiveness of approaches to the conservation process, as variations in pigment composition, mortar structure, and exposure conditions can lead to differing degrees of degradation and require tailored conservation strategies [1].

**Figure 1:**
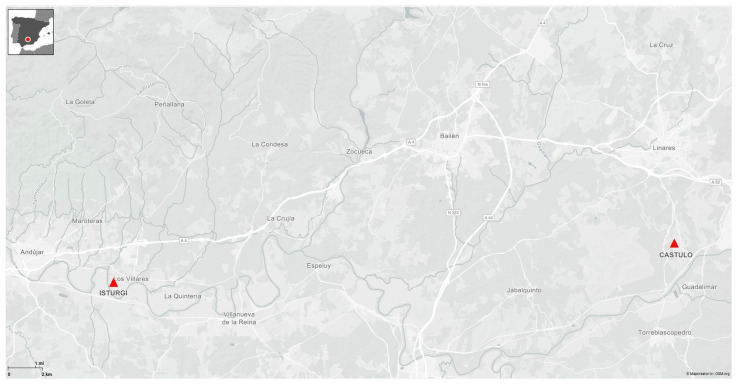
Aerial location mapping of the Cástulo and Isturgi sites.

Two significant archeological sites in the province of Jaén (Spain) preserve distinctive sets of Roman mural paintings, highlighting the unique conservation challenges these artworks present. The wallpainting from the Sala del Mosaico de los Amores at the archeological Site of Cástulo and those from the Iberian–Roman villa of Isturgi have a shared decorative style and similar chronology, both dating to the 1st and 2nd centuries AD (Figure 1). In both archeological sites, the mural coatings follow a decorative scheme composed of large red panels separated by interpanels, a common design across the Roman Empire. In the case of the archeological Site of Cástulo, the interpanels are adorned with metallic candelabras and figurative and vegetal decorations. In the Iberian–Roman villa of Isturgi, these divisions are created by columns, a motif also found in significant works such as the mural paintings from *Calle Añón* in Caesaraugusta, dated to the late 2nd century AD Caesar Augusta (Zaragoza), and the decorative programme of the triclinium [2].

In both cases, the paintings were found collapsed within the rooms. The murals at the Archaeological Site of Cástulo were discovered in 2011, while those from the Iberian–Roman villa of Isturgi were uncovered in 2018. Although remains typically found in excavations are often limited to fragments that rapidly degrade due to biological agents, mineralogical processes, and adverse climatic conditions, these fallen mural paintings are of significant value due to their size and formal variety. They offer crucial information about the decorative style of the rooms, their chronology, and even the factors contributing to their deterioration [3]. This study focuses on the large red panels, which are characteristic of the decoration in opulent Roman villas. The study of the red panels, which are characteristic of the decoration in opulent Roman villa, is essential not only for understanding the decorative styles and artistic conventions in opulent Roman villas but also for guiding restoration and conservation processes. Both panels are particularly notable due to their high-quality burnished surface. However, their conservation state differs markedly, with the fragments recovered from Cástulo exhibiting more severe degradation and instability compared to those from Isturgi. Red is a highly significant color in Roman wall paintings, with a broad palette of red pigments in use. Among these, lead tetroxide, Pb_3_O_4_, referred to as *Minium secundarium* by Plinio, stands out as one of the few bright red colours available on the market [1]. Cinnabar/vermilion (mercury sulphide, HgS), also known as *Minium* in Roman times, was another prominent red pigment [2,3]. Romans often applied wax to protect it, as it could darken to brown upon degradation. Another widely used red pigment was red iron oxide (Fe_2_O_3_), denominated *Hematites*, which has been employed since antiquity [4]. These pigments were sometimes mixed. This was often the case with cinnabar/vermilion and hematite, to reduce the high cost of cinnabar/vermilion and to mitigate its darkening when exposed to light [4,5].

The application of characterization techniques to materials present in mural paintings, including the pigments in the Roman palette, has helped to identify and understand their evolution. Their morphological, chemical, electronic and structural properties can be analyzed using microscopy, spectroscopy, and elemental analysis, among other methods [5]. Different variations of microscopy have allowed for the morphological analysis of a multitude of materials, and energy-dispersive X-ray spectrometry is one of the most used techniques for the identification of the compounds in the Roman palette [6,7,8]. Common pigments that were identified include red and yellow ochre, Egyptian blue, carbon black, and lime/chalk white [9,10]. In this study, the analysis of red pigments is highly relevant, as they dominate the large spatulated backgrounds of the pictorial compositions in the mural paintings at both archeological sites.

These structures were also analyzed using multi-technology approaches, including FTIR, Raman, XRF, XRD, portable instruments (when technologically possible), and traditional laboratory equipment after taking micro-samples [8,10,11]. These analytical techniques enable a detailed examination of the physical and chemical characteristics of pigments, facilitating an understanding of both their original composition and their alterations over time due to environmental exposure and chemical degradation. Such insights are essential for developing conservation strategies that preserve the original material’s integrity and ensure the long-term stability of these paintings.

The study of red pigments in Roman mural paintings has been widely documented, highlighting the sophisticated understanding that Roman artists had of materials and their properties [1]. Through the use of iron oxides (such as hematite), mercury sulphide (cinnabar/vermilion), lead-based reds, and other compounds [12,13,14], Roman artists were able to achieve a broad spectrum of red hues, showcasing not only their expertise in colour production but also their advanced technical skill in the preparation and application of these materials. Analytical studies of these pigments reveal that Roman artists employed varied techniques and knowledge to manipulate pigment stability, achieve specific chromatic effects, and adapt their methods to different types of surfaces. Furthermore, the ability to select and process these mineral sources reflects a comprehensive understanding of both local and imported materials, suggesting that Roman painters were adept in resource selection and had access to trade networks supplying diverse pigment materials [15,16,17].

Most studies focused on the identification of the compounds that generate the desired colour; their characterization techniques can also provide evidence of the evolution of the structures, which would allow us to understand the degradation processes of the structures and offer alternative techniques for their conservation.

This contribution focuses on the study of red panels from the aforementioned prominent archeological sites in the province of Jaén: the Archaeological Site of Cástulo and the Iberian–Roman villa of Isturgi. The contribution presents an approach that includes contextualizing the red panels, examining the techniques used in their creation, analyzing their current condition, and identifying their conservation status in chemical, electronic, and structural terms. The interdisciplinary study of Roman mural pigments offers a unique perspective of the technological capabilities and cultural practices of ancient artisans, serving as a bridge between art history, archeology, and conservation science. The findings from Cástulo and Isturgi contribute significantly to our understanding of Roman pigment technology and provide a methodological framework that can be applied across different archeological sites to investigate regional material variations and sourcing practices. Future research might focus on extending these techniques to other hues in the Roman palette and comparing pigment compositions across a broader geographic range within the Empire, illuminating potential trade routes and regional differences in pigment use.

## 2. Materials and Methods

### 2.1. Archeological Site of Cástulo

Red samples were analyzed from the Archaeological Site of Cástulo, specifically from the red panels in the Hall of the Mosaico de los Amores. This room was discovered in 2011, and its paintings are currently undergoing restoration at the University of Granada as part of the FORVM MMX and Cástulo Siglo XXI projects. Cástulo, founded in the third millennium BC, is situated in the heart of a mining region among the municipalities of Linares, Lupión, and Torreblascopedro in the province of Jaén (Spain). The city reached its zenith during the Iberian–Roman period, establishing itself as one of the most significant settlements on the Iberian Peninsula [18,19].

**Figure 2:**
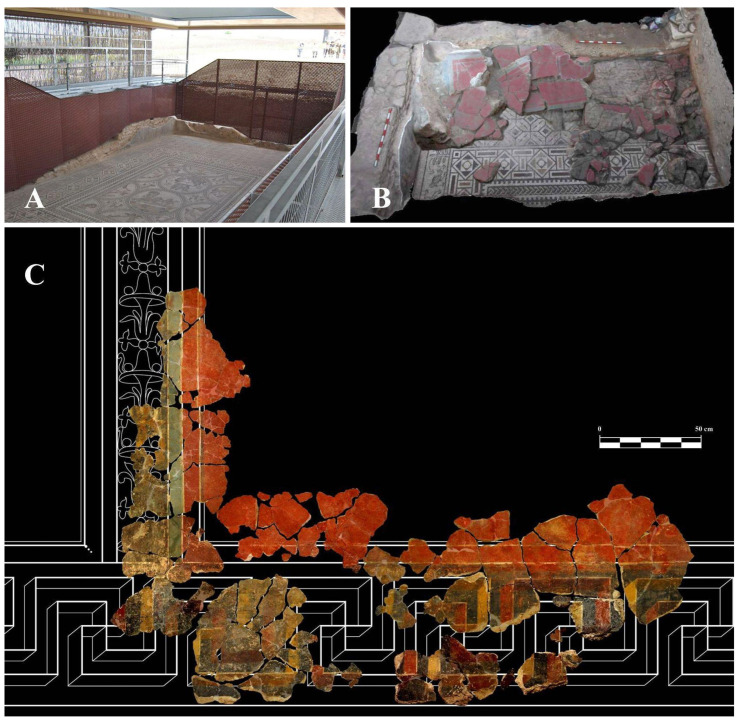
(**A**) General view of the current state of the Hall of the Mosaico de los Amores. (**B**) Photogrammetry taken at the time of its discovery, showing the remains of large red panels in a possible intentional collapse over the pavement. (**C**) Digital reconstruction of the restored panels based on the decorative scheme.

Among the identified public buildings, Building D stands out. It features a series of courtyards and rooms adorned with mosaics and wall decorations, dated to the 1st and 2nd centuries AD. Notably, the Hall of the Mosaico de los Amores (also called Room 1) is dated between the late 1st century and early 2nd century [20]. This hall measures approximately 6 m × 12 m and contains an extraordinary mosaic that gives the room its name, along with rich wall decoration (Figure 2A). As can be seen in Figure 2B, at the time of its discovery, most of the wall coverings were documented on the floor due to intentional collapse, with only a small portion of the dado remaining in situ. Fortunately, many fragments of mural paintings have been preserved, demonstrating the exceptional skill of the artists who created them [20]. The restoration of these mural paintings at the University of Granada has revealed their decorative scheme (Figure 2C). This scheme follows the typical Roman tripartite layout, dividing the wall into three sections. The lower section features a skirting board with a black background and a gamay cross in perspective. The middle section includes large red panels, framed with a double border and accented with dots at the corners. These panels are separated by interpanels decorated with metal and vegetal candelabras, framed by bluish-green bands and white fillets, as shown in Figure 2. The upper section features a relief cornice adorned with delicate ornamental and floral motifs [19].

### 2.2. Iberian–Roman Villa of Isturgi

The archeological site of Isturgi is located in the western Vega of the Guadalquivir River, in the province of Jaén (Spain). The Los Villares site is about 5 km east of Andújar and 1 km west of the town of Los Villares. This location was an important centre for the production of Hispanic terra sigillata, with significant economic activity during the 1st and 2nd centuries AD. It corresponds to the city “*Municipium Isturgi Triumphale*” mentioned by Pliny in his Natural History [21].

**Figure 3:**
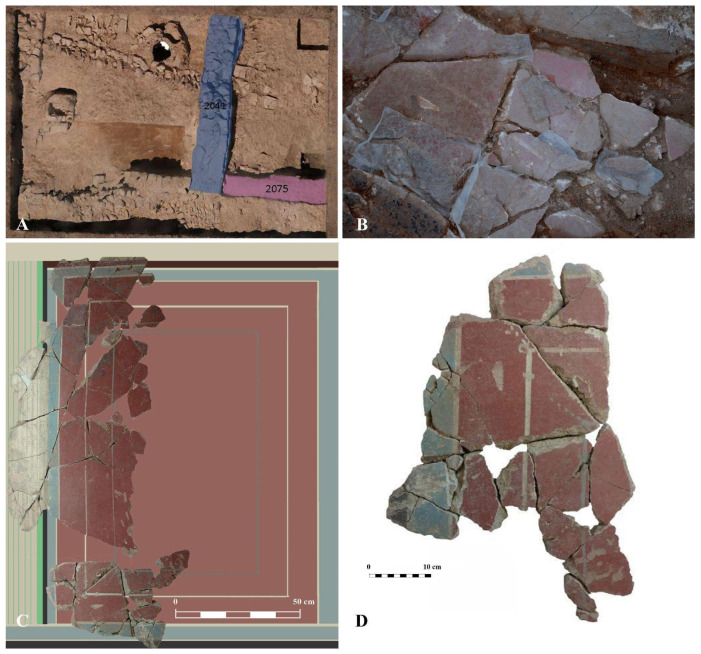
(**A**) General view of the Isturgi site indicating walls 2041 and 2075, from which the remains of mural paintings were obtained. (**B**) Detail of the extracted mural paintings. (**C**) Digital hypothesis of the restored PANEL 1. (**D**) Photographic documentation of PANEL 2 after its cleaning intervention.

The mural painting fragments studied in this work come from one of the surveys conducted in 2018. Chronologically, these coatings belong to the construction and occupation phases of the early and mid-imperial periods. Specifically, the studied panels come from wall MR2041 (Figure 3A), which features a plain decoration in red, blue, and black, with white filets separating the three colours. The decorative scheme and technical quality of the paintings, as illustrated in Figure 3B–D, are very similar to that of those identified at the Archaeological Site of Cástulo.

### 2.3. Sample Collection

At the archeological sites of Cástulo and Isturgi, both renowned for their wall paintings, a similar procedure was employed for pigment sampling. The sites were divided into relevant sectors, and stratified random sampling was used to select 20 points where wallpainting with visible red pigment traces was present. Each sampling point was meticulously documented with high-resolution photography and detailed sketches, while noting contextual information such as the condition of the wallpainting and pigment degradation. The sample nomenclature was identified using the abbreviation “CA” for mural painting samples from the Archaeological Site of Cástulo, and “IS” for mural painting samples from Isturgi. The samples used in this study are part of the comprehensive documentation collected during the entire intervention process. Selections were made from pieces where the paint and associated zones were identified but were not structurally contextualized, minimizing the impact on the restoration process. The use of Paraloid B-72 during the extraction process in the archeological sites was also documented, and this information was considered during the characterization of the samples. Samples were carefully extracted using fine scalpels and micro-sampling tools to minimize damage, placed in sterile containers, and stored in a cool, dry environment. The samples were transported under controlled conditions, catalogued, and prepared for analysis according to standard protocols.

### 2.4. Characterization of the Samples

Several microscopy techniques were employed to analyze the morphology of the samples in detail.

#### 2.4.1. Stereoscopic Microscopy

The first stage of the sample analysis involved a stereoscopic examination of the unprepared sample in various orientations. This initial step requires only a small amount of the sample and is crucial for selecting the most appropriate methods for the next phases of the study. It helps identify the most representative and relevant areas. This technique provides a preliminary analysis, allowing for the observation and identification of the paint layers and the morphology of the mortar. Stereoscopic microscopy was performed using a NIKON SMZ 1000 microscope, (Faculty of Fine Arts, University of Granada, Granada, Spain) allowing for detailed microphotographs of both the surface and the cross-section.

#### 2.4.2. Optical Microscopy

This type of analysis requires the preparation of extracted samples. For this phase of the study, the samples were processed as polished thin sections. These sections were obtained from cross-sections of the samples, which were mounted on a slide—typically made of methacrylate—and subsequently polished to achieve a thin layer of only a few microns thick. Once prepared, these thin sections can be examined under reflected, transmitted, and combined (reflected + transmitted) light microscopy.

This analytical technique allows for the study of the stratigraphy and thickness of the pictorial layers, as well as the determination of the size, morphology, and colour of the pigment particles. Regarding pigment identification, this method enables the observation of key optical properties, such as pleochroism, refractive index, Becke line, relief, and birefringence, facilitating the preliminary identification of some pigments.

For this analysis, Carl Zeiss-Jena Jenalab and Olympus BX-0 microscopes (CIC, University of Granada, Granada, Spain) were used. The latter is equipped with a DP-20 microphotography system, enabling the extensive documentation of all samples through microphotographs. In both cases, observations were conducted under plane-polarized and cross-polarized light. Both microscopes are housed in the Department of Mineralogy and Petrology at the University of Granada.

#### 2.4.3. Scanning Electron Microscopy (SEM)

After being examined under optical microscopy, the samples were coated with a thin layer of carbon in the sample preparation laboratory of the CIC at the University of Granada to enable their analysis using scanning electron microscopy. Scanning electron microscopy (SEM) and energy-dispersive X-ray spectroscopy (EDX) (CIC, University of Granada, Granada, Spain) were conducted using Leo Gemini 1530 and Leo 1430 VP microscopes, both equipped with Inca 350 version 17 EDX systems from Oxford Instruments. Point microanalyses were performed with a filament current of 500 pA, a beam energy of 20 keV, and a spectral resolution of 10 eV/ch, while X-ray maps were acquired using the Leo 1430 VP with a filament current of 1 nA and a resolution of 20 eV/ch. The crystalline structure was analyzed using powder X-ray diffraction (XRD) in a Bruker D8 Discover device working with a Cu Kα radiation source (λ = 1.5406 Å), equipped with a Pilatus3R 100 K-A detector, which was registered within a 2θ range of 6–80° at a rate of 0.04° min^−1^. The software QualX^®^ and the Crystal Open Database (COD) were used to identify the crystal phases.

#### 2.4.4. The Fourier Transform Infrared (FTIR)

The Fourier transform infrared (FTIR) (Faculty of Science, University of Granada, Granada, Spain) spectra were taken with a Spectrum 65 device from Perkin–Elmer, which could measure absorbance within the range of 550–4000 cm^−1^. This was equipped with a compact rotary interferometer which does not require correction for misalignment and a high-performance, room-temperature, standard MIR detector. The samples were measured as powder, and the Spectrum software was used for the analysis of the spectra.

#### 2.4.5. X-Ray Photoelectron Spectroscopy (XPS)

X-ray Photoelectron Spectroscopy (XPS) (Faculty of Science, University of Granada, Granada, Spain) was applied to study the elemental composition at the surface of materials in a Kratos AXIS UltraDLD device operating using an X-ray source from Al Kα. The spectra were referenced to the C1s peak of adventitious carbon at 284.6 eV. The software XPSpeak 4.1^®^ was used for the deconvolution of peaks with Shirley background correction.

## 3. Results and Discussion

### 3.1. Analysis of the Techniques Used in the Roman Mural Paintings

The samples studied correspond to the fresco technique, a method that involves the fixation of pigments through the carbonation of lime. This technique was of great importance in Roman times, as it was widely used to decorate walls and ceilings in public buildings, private homes, and monumental structures. The durability and striking colours achieved through this method made it a preferred choice for Roman artists and architects. For this, the pigments are applied, diluted in water, to the still-wet lime plaster. The calcium hydroxide from the wet lime mortar, upon contact with the carbon dioxide in the air, forms a film of calcium carbonate that ensures the adhesion of the colours to the substrate. In most of the samples, the paint layer was firmly adhered to the preparation layer, with no observed discontinuities, and a single carbonation layer was present at the top of the stratigraphic sequence. This indicates that the paints were applied to a fresh surface, confirming that the fresco technique was employed [1,22]. Some areas of decoration in the analyzed mural paintings, such as details in blue, green, or white, found in overlapping decorative layers, may have been executed using the secco technique with an organic binder or through a second carbonation of the surface during the burnishing process, as was common in this technique. This practice has been documented in Roman wall paintings, where pigments unsuitable for true fresco, such as Egyptian blue or lead white, were often applied via secco [1]. Additionally, studies on Roman painting techniques indicate that a secondary carbonation process could occur due to the mechanical action of burnishing, further fixing the pigments to the surface.

**Figure 4:**
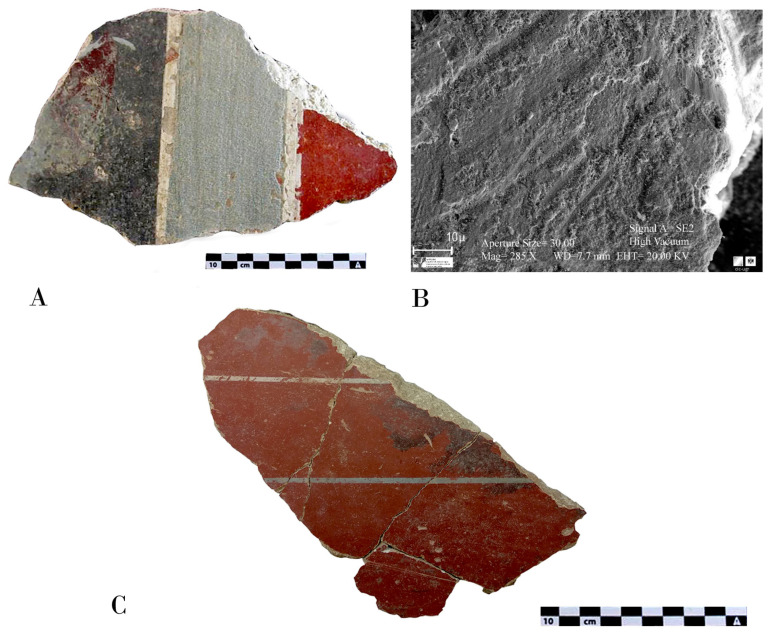
(**A**) Fragment from the Archaeological Site of Cástulo showing the difference between the burnished surface (red) and the unburnished application (blue). (**B**) High-resolution scanning electron microscopy image of a sample taken from the same fragment, revealing the burnishing marks on the surface. (**C**) General image of one of the restored fragments from Isturgi, showing the polished surface.

Both the fragments obtained from Isturgi and Cástulo exhibit a smooth and waxy finish on the red backgrounds, likely resulting from the burnishing of the pictorial surface. This burnishing is related to the term “politiones” described by Vitruvius, which involved polishing the surface with a hard instrument to facilitate the release of water and calcium hydroxide, promoting carbonation on the surface and reinforcing colour fixation. This technique also imparts a very characteristic shine that can be observed in these fragments and is typical of Roman wallpainting throughout the empire. The use of this technique reinforces the idea that these paintings were created following the fresco method described by classical authors, where plaster is polished before receiving the decorative motifs and after applying the pigments. In this way, some underlying moisture is drawn to the surface, enabling the creation of the fresco paintings, preserving the surface’s plasticity for a much longer time, and making brushstrokes and workday joints much less visible (Figure 4A–C) [3].

**Figure 5:**
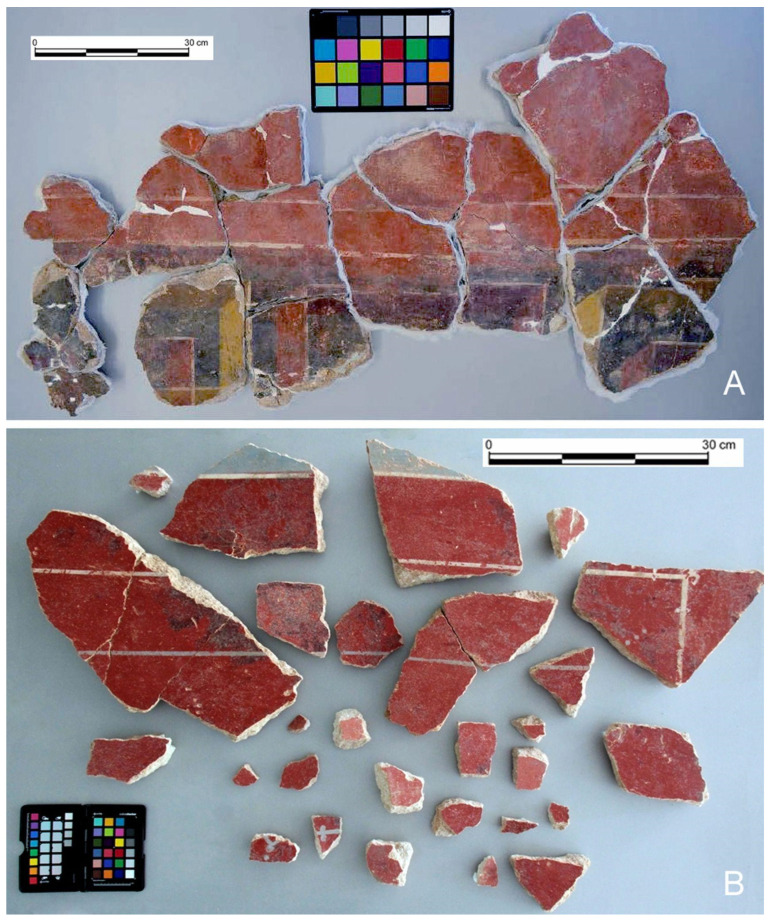
(**A**) General view of a set of mural painting fragments from the Archaeological Site of Cástulo, showing the uneven condition of the red backgrounds. (**B**) General view of a set of treated mural painting fragments from the Ibero-Roman villa of Isturgi, showing the exceptional preservation of the burnished red backgrounds.

Regarding the conservation state of the red pigments, both sites contained a significant number of preserved fragments. In the case of Cástulo, the red backgrounds were in a heterogeneous degradation state, with multiple areas presenting an abundance of carbonate crusts that are difficult to remove. These encrustations may also enhance the degradation of the paintings. The cleaning processes carried out in the restoration laboratory revealed the complexity of removing these deposits without damaging the original colour layer, which is quite fragile. Nevertheless, sections of the backgrounds with incrustations were effectively cleaned. Additionally, several sections without carbonate deposits were identified and extracted. In contrast, the fragments from Isturgi did not present this issue. The extracted fragments were in good condition, with superficial concretions and dirt that could be easily removed using physical methods, without affecting the paint layer, which showed a high-quality burnished surface, as presented in Figure 5A,B. The structural analysis was performed on samples with relatively low (negligible at a macroscopic level) levels of encrustation, exhibiting a degree of deterioration representative of the extracted fragments. This allowed for the assessment of the significance of structural differences and their potential relationship with the state of degradation.

Both paintings exhibit common stylistic motifs corresponding to the Third and Fourth Pompeian Styles. In the case of Cástulo, as specified in a previous contribution [18], these paintings correspond to the Third Style (referred to as ornamental) from the first half of the 1st century AD and the Fourth Style (considered theatrical) developed from the second half of the 1st century AD [23].

**Figure 6:**
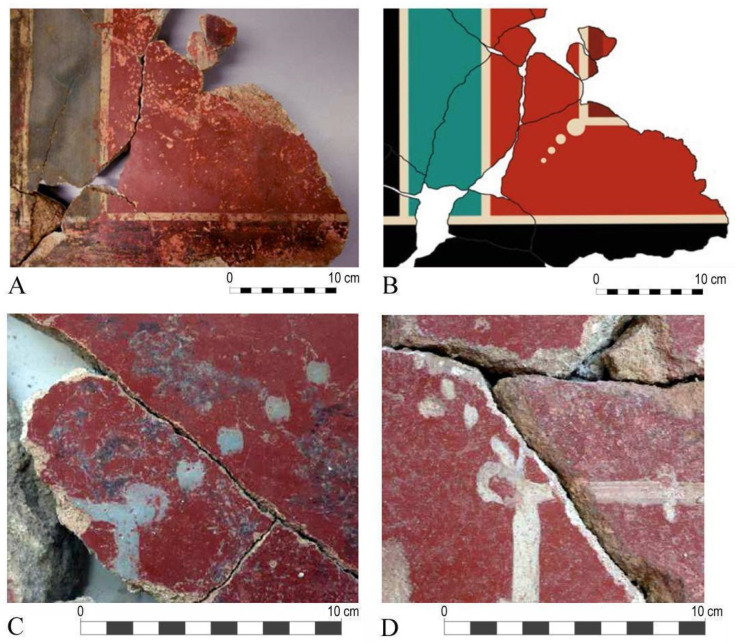
(**A**) Preserved remains of the points at the angles in the framing strokes in Cástulo. (**B**) Graphic reconstruction of the points at the angles in the framing strokes in Cástulo. (**C**) Blue-coloured detail of the same motif in the mural paintings of Isturgi. (**D**) Detail of the white-coloured decoration in the wall paintings of Isturgi.

In terms of decorative features, the recovered mural fragments exhibit a distinctive element: dots at the corners of the framing lines. This characteristic originated in the so-called Third Pompeian Style. As this decorative motif spread to provincial painting, it evolved into successive series of dots or ornamental floral elements. In Spain, this motif is present in mural paintings from sites such as Baetulo, Celsa (in the House of Hercules), Tiermes, Bilbilis, and Caesaraugusta (in the Forum) [23]. As can be seen in Figure 6A,B, this decorative element is also identified in the fragments from Cástulo, where dots appear in two corners of the panels, with a wide horizontal band and a narrower vertical band, both in red. A similar decorative feature is found in the mural paintings from Isturgi, on wall MR2041, where the dots are depicted in blue and white (Figure 6C,D). The decorative similarities between both archeological sites may suggest that the paintings were created by the same group of artists. This hypothesis is further reinforced by their geographical proximity within the province of Jaén and the similar chronological timeframe in which the paintings were dated.

### 3.2. Contextualization of Roman Red Pigments

To analyze the red panels, this section provides an analysis of the Roman red palette, contextualizing it based on the study area. The colours used in Roman wall paintings were classified by Pliny based on their brightness and chromatic intensity. He referred to pigments with vivid tones as *floridi*, distinguishing them from the common pigments, called *austeri*, meaning austere or dark. The bright or *floridi* colours, provided to the painter by the patron, included *Minium*, *Armenium*, cinnabar/vermilion), *Chrysocolla*, indigo, and *Purpurissum*. The other colours were dark. Based on this classification [1], a theory which suggests that this organization distinguished between translucent and opaque colours was developed. In cases of layered application, the *floridi* colours would occupy the uppermost layer. Among the red pigments used, lead tetroxide, known as *Minium secundarium* by Pliny, stands out as one of the few bright red colours available in trade. Ancient texts describe its production process, passed down by various authors. Pliny mentioned that its discovery was accidental, following the Piraeus fire, while Vitruvius also recorded that it “was discovered as the result of an accidental fire”. Pliny noted two additional varieties in his writings [2]. Lead red was used in Roman wall painting, although its application in the fresco technique is controversial due to its instability in alkaline environments. Analytical studies have shown that, in some cases, minium was mixed with more stable pigments, such as iron oxides, or applied in surface layers with a possible secco fixation using organic binders. Furthermore, its alteration has been documented in ancient frescoes, where it darkened due to chemical reactions with environmental sulphides [1]. These findings suggest that, although minium may have been applied in fresco, its long-term stability depended on the environmental conditions and the painting technique used.

Regarding the extraction of this mineral in the Roman Empire, it is likely that it could have been sourced from the mining region of Cástulo, known for its silver and lead deposits. Pliny the Elder references this in *Naturalis Historia* (XXXIII, 40, 118trad. Rackman: 89) [18], noting the extraction of this pigment in Sisapo, a former Roman municipality within the pre-Roman region of Oretania, of which Cástulo was the capital. This corresponds to the present-day village of La Bienvenida, in Ciudad Real.

The most widely used red pigment in Roman times was red iron oxide, known as *Hematite*, a natural pigment that has been in use since antiquity. Hematite is probably the most-used red pigments in wall paintings, not only in Roman wall paintings but also in other historical periods. It was frequently employed in Roman wallpainting, particularly for large red panels. This pigment has been identified in several locations, including the mural painting in the Vesuvian area [15]. In the Iberian Peninsula, it was found in numerous archeological sites, such as the murals of Bílbilis, El Ruedo in Almedinilla, the Roman necropolis of Camino Viejo de Almodóvar, and Guadix [5,6,7], among others.

In Roman wall paintings, three types of hematite are distinguished based on their crystallization. The first type, a well-crystallized red iron oxide, can have various shades, including blood red, ochre, or brown. This corresponds to the pigment called *Sinopis* by Pliny (XXXV, 13). The second type of hematite is associated with quartz, plagioclase, potassium feldspar, illite, and kaolinite, and matches Pliny’s description of *Rubricae* (XXXV, 15). The third type of hematite is produced by the dehydration of goethite when exposed to temperatures above 850–900 °C [24].

Hematite is often mixed with other pigments to modify their properties. For example, it was found combined with lead red. This pigment had to be manufactured specifically through the calcination of white lead; it is extremely rare naturally. This means that it would be much more expensive than haematite, which occurs naturally and is very common. Therefore, the main reason for mixing minium and haematite was probably cost, in addition to improving the colour [25]. Cinnabar/vermilion) (HgS), known as *Minium* in Roman times, is worth mentioning and, according to authors like Vitruvius, was obtained from mines in Almaden (Ciudad Real, Spain) [26,27]. Despite its high cost and conservation issues, such as darkening due to light exposure, it was one of the most commonly identified pigments, both alone and in mixtures, in paintings from this period. The references by Pliny and Vitruvius are the richest sources regarding the incorporation of cinnabar/vermilion into the palette of Roman painters. Both authors emphasize its status as a highly valued product, with its usage dating back to ancient Rome, particularly in sacred contexts. Pliny (Nat. 33. 111) refers to earlier reports of the practice of colouring the face of the statue of Jupiter and the bodies of generals in triumphal parades with *Minium* [10,28]. In Hispania, its widespread use is notable in the paintings of Bílbilis (II style paintings in Domus 2 and III style in Domus 3, Augusta Emerita (Casa del Mitreo), and in the river port of Caesaraugusta [29]. As mentioned earlier, it was common for this pigment to be mixed with iron or lead red to reduce costs or achieve tonal gradation. In addition to mixing pigments, as previously mentioned, layering pigments was common in Roman times. This technique has been described by Pliny and demonstrated in various analytical studies. The process involves applying a low-cost pigment as the base (usually a red or yellow iron oxide), over which a layer of a more expensive pigment, such as cinnabar/vermilion or lead red, is applied. This method aimed to reduce costs without affecting the final appearance [29]. Cinnabar/vermilion has been identified in successive layers at sites such as the Monte d’Oro Area (Rome) or Blanes Dump (Mérida), among others.

### 3.3. Characterization Results

**Figure 7:**
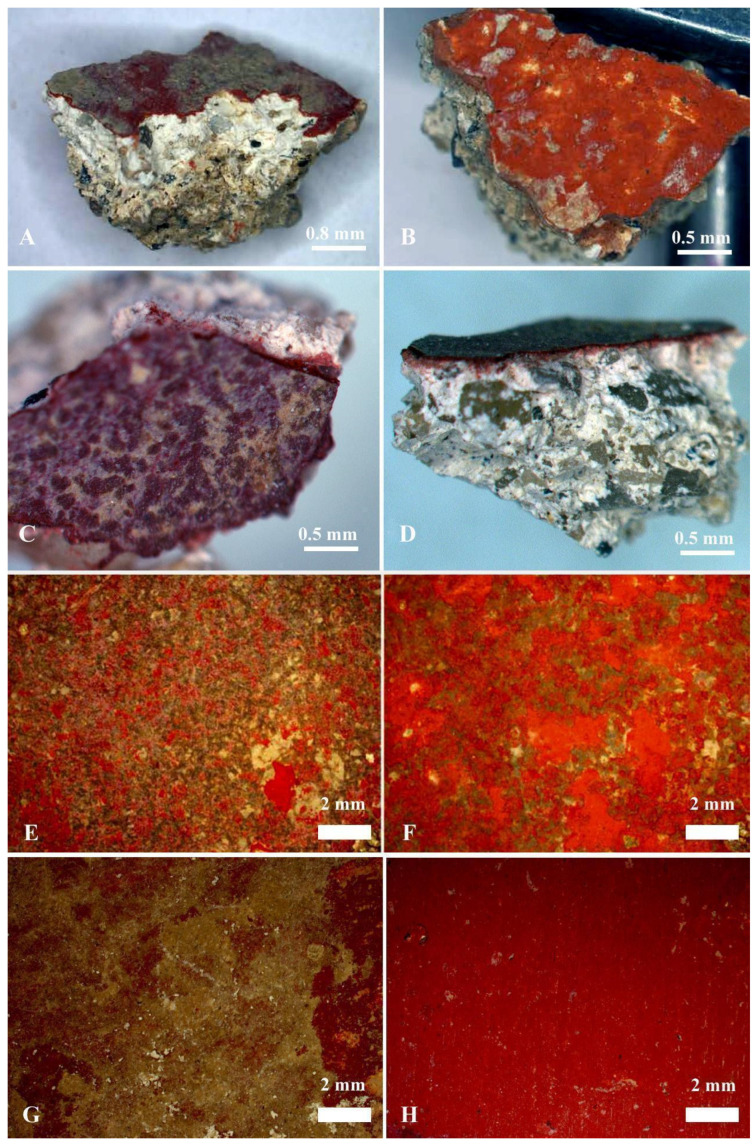
Stereoscopic micrographs of representative red samples from Cástulo (**A**,**B**) and Isturgi (**C**,**D**). (**E**) Condition of the pictorial layer of the mural paintings of Cástulo before cleaning, where a large accumulation of deposits obscures the original colour. (**F**) Condition of the pictorial layer after cleaning. (**G**) Condition of the pictorial layer of the mural paintings of Isturgi before cleaning, where a high accumulation of carbonate concretions is observed. The cleaning method used for both fragments was a physical procedure involving a scalpel and a fibreglass pencil. (**H**) Condition of the red pictorial surface of Isturgi after cleaning. In this image, the high quality and good state of preservation of the red pictorial layer in this mural can be observed.

Representative samples were selected for the study to analyze Roman paintings from Cástulo and Isturgi sites using stereoscopic microscopy. The three-dimensional view provided by this technique facilitates the identification of the layered paint structure, which would not be as apparent when using other kinds of microscope. This type of analysis also allows for the examination of the plaster composition. As shown in Figure 7A–D, in the case of Cástulo, a clearer layering is evident, with a final layer of very white mortar using aggregates of low granularity. In contrast, in the case of Isturgi, the layering where the grain size decreases is not as distinct, with relatively large aggregates present near the pictorial surface. Panels presented in Figure 7E,F from Cástulo show the detailed surface features and the underlying stratigraphy of the paint layers. The stereoscopic images show evidence of pigment accumulation and notable ageing effects, such as cracking. Additionally, a pronounced sign of delamination was present in the red paint layer, which showed more extensive areas of detachment compared to the Isturgi samples. Figure 7G,H from Isturgi generally exhibit a lower level of deterioration. The stereoscopic analysis reveals fewer visible delamination areas, indicating a more stable paint layer structure compared to Cástulo. Additionally, the difference in the shade of red between the two sites suggests variations in the pigment formulation or degradation processes. These colour differences, along with the observed stability of the paint layers in Isturgi, provide insights into historical pigment use and the potential environmental impacts affecting the artwork.

**Figure 8:**
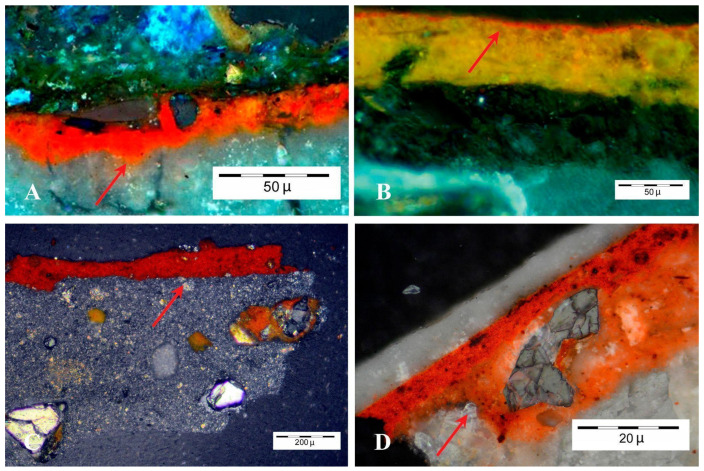
Optical microscopy images of samples prepared in thin sections from the mural paintings of Cástulo (**A**,**B**) and Isturgi (**C**,**D**).

In addition to the stereoscopic microscope analysis, further examination of the thin sections provided additional insights into the composition and structural integrity of the paint layers. Figure 8A,B correspond to profiles visualized using optical microscopy, respectively, for two selected samples from Cástulo. Figure 8C,D are representative of the paintings collected from Isturgi. The thin sections allowed for a more detailed assessment of the stratigraphy and interaction between the different paint layers. This method revealed subtle variations in layer thickness and composition that were not as easily discernible with stereoscopic microscopy alone. The detailed examination highlighted specific areas where the paint layers exhibited different adhesion properties, which may be indicative of variations in the painting techniques or materials used at different periods.

The samples observed under optical microscopy show that red paint was used as a base for the entire painting, and this base layer exhibits variable thickness. In the case of Cástulo, Figure 8A shows such a red base overlaid with an additional pigment, Egyptian blue, a well-known ancient pigment made from copper silicates, as noted below, based on elemental analysis by scanning electron microscopy [17,30]. In the image, the Egyptian blue crystals can be seen overlaying the red background paint layer, as was common in Roman wall paintings. In the samples from Isturgi (as observed in Figure 8C,D), the analyzed red layer exhibits significant consistency and thickness. In Figure 8C, the red layer shows considerable thickness and high homogeneity. On the other hand, in Figure 8D, the application of a superimposed white pigment can be observed, corresponding to one of the decorative bands, probably applied using the secco technique or during a second spatulation of the surface. This white pigment is whitewash (denominated cerussa) a white pigment commonly used in Roman times (Figure 8D) [31]. This use of red as a foundational layer, along with the variable thickness and different additional pigments, illustrates the varied techniques and materials employed in Roman painting practices across the two sites. On the other hand, the analysis using optical microscopy focused on characterizing the exposed red layers. The average thickness measured was approximately 10–40 µm, which is typical for Roman fresco paintings. In general, the red layers obtained from Isturgi (Figure 8C,D) show greater uniformity in thickness compared to those from Cástulo (Figure 8A,B). This greater uniformity in thickness is associated with the better preservation of the Isturgi samples, which exhibited less surface flaking compared to the more deteriorated Cástulo samples.

**Figure 9:**
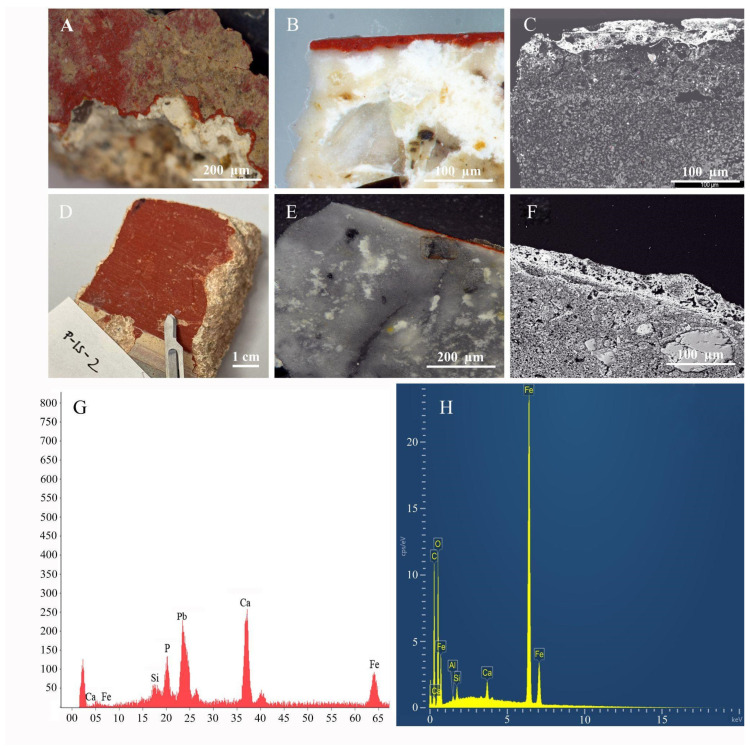
Example of the methodology used for the study of samples from Cástulo and Isturgi. Top side: (**A**) microscopy image of the unprepared sample (Castulo); (**B**) optical microscopy image of a thin section (Castulo); (**C**) scanning electron microscopy image (Castulo). Bottom side (Isturgi): (**D**) sampling procedure using a scalpel (Isturgi); (**E**) optical microscopy image of a thin section (Isturgi); (**F**) scanning electron microscopy image (Isturgi); (**G**) SEM spectrum of the analyzed red pictorial layer (Castulo); (**H**) SEM spectrum of the analyzed red pictorial layer (Isturgi).

Stereoscopic and optical microscopy analyses of samples taken from the Isturgi and Cástulo (Figure 9) fragments were complemented by the preparation of thin sections and their metallization for examination using scanning electron microscopy. The identification of specific pigments through optical microscopy analysis can be further validated through scanning electron microscopy (SEM), which provides detailed morphological information, and through elemental analysis, which detects key chemical elements such as Pb, Si, Cu, or Fe, among others, depending on the composition of the pigments. This multi-analytical approach enhances the accuracy of pigment characterization, offering critical insights into their composition, stratigraphy, and potential degradation processes [32]. As demonstrated in Figure 9, the analyses showed the use of a high-quality red layer. In the case of Cástulo, this layer was applied over a well-differentiated final stratum of high purity, featuring fine aggregates. In contrast, in Isturgi, larger aggregates were identified near the surface. The elemental analysis of the red pigments from Isturgi indicated a predominant presence of Fe, suggesting the use of iron oxide pigment for the red background, known in Roman times as hematite, a natural pigment that has been in use since antiquity [33,34]. In Cástulo, the analysis revealed a predominant presence of Pb in the red layer, along with other elements such as P, Si, Ca, and Fe. The analyses suggest the presence of two red pigments: iron red and lead red. Layering pigments was common in Roman times. This technique was described by Pliny and demonstrated in various analytical studies [35]. The process involves applying a low-cost pigment as the base (usually a red or yellow iron oxide), over which a layer of a more expensive pigment, such as cinnabar/vermilion or lead red, is applied. This method aimed to reduce costs without affecting the final appearance [36].

**Figure 10:**
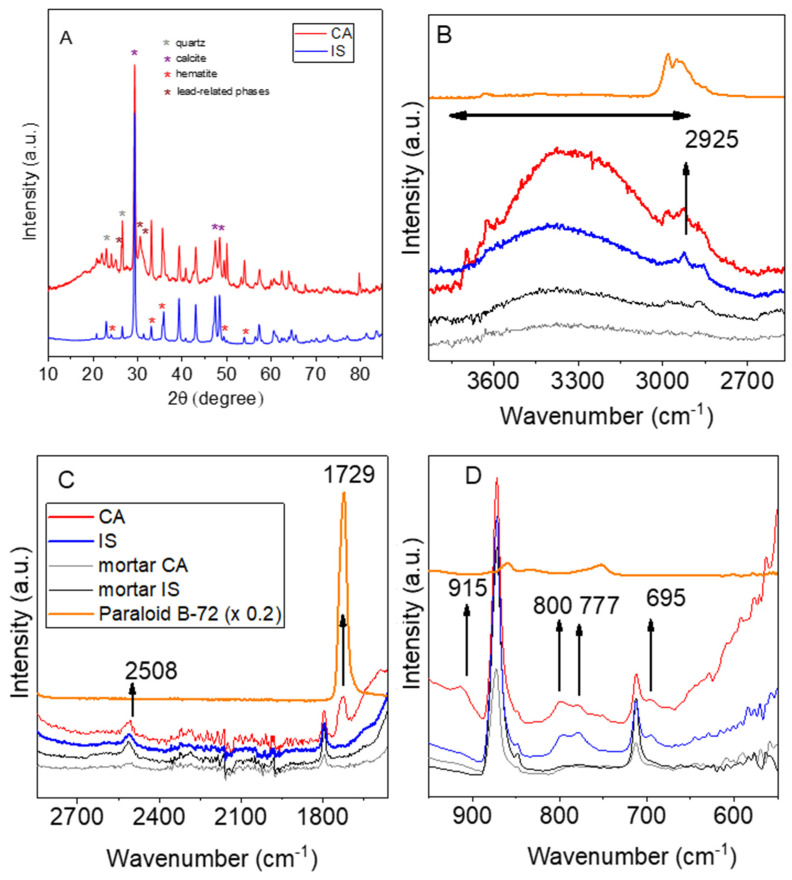
(**A**) XRD patterns of representative samples from Cástulo (CA) and Isturgi (IS). (**B**–**D**) FTIR spectra of the red samples obtained from Cástulo (CA) and Isturgi (IS), and the base mortar on which the pictorial layer was applied. FTIR spectra obtained for Paraloid B-72 are also included (**B**–**D**). The colour code (legend) for Figures (**B**–**D**) is the same and as that in Figure (**B**). a. u. (arbitrary unit).

Figure 10A shows the diffractograms of samples obtained from representative areas of the red panels of Cástulo and Isturgi. Clear differences were observed between the XRD results of Isturgi and Cástulo. In the Isturgi sample, well-defined peaks allowed for the precise identification of hematite (COD 9000139), with characteristic peaks at 2θ = 24.1°, 33.2°, 35.6°, 49.5°, and 54.0° [37]. Calcite (COD 9009668), with relevant peaks at 2θ = 29.4° and 48.5° [37], and quartz (COD 9010146), with prominent peaks at 2θ = 20.9° and 26.6° [38], were also identified and can attributed to the commonly used mortar support. The Cástulo sample exhibited a more complex diffractogram, including crystalline components (hematite, calcite, and quartz), as well as additional peaks, which could be associated with lead species. Peaks at 2θ = 26.3° within the 30–32° range suggested the potential presence of lead red (COD 9013444) [39], though in small proportions. The diffractogram obtained from the Cástulo sample suggests that the initial structure of lead red may have evolved into other phases (e.g., sulphates). This structural change is attributed to the reactivity of lead red in the presence of atmospheric agents and humidity, conditions commonly found in the preservation environment of ancient wallpainting. In this context, the transformation of Pb_3_O_4_ into secondary minerals, such as lead sulphates and halides, is a plausible and frequent process in Roman-era works [40]. We cannot rule out the presence of other species, such as iron plumbates, in low concentrations.

More information about the structure of representative red samples obtained from Cástulo and Isturgi can be gathered using Fourier Transform Infrared Spectroscopy (Figure 10B,D). The spectral data reveal a complex array of absorption bands indicative of various chemical constituents and potential conservation treatments. A broad band ranging from 3700 to 2800 cm^−1^ was observed across all samples, with a particularly pronounced presence in the CA sample. This band is characteristic of O-H stretching vibrations, suggesting the presence of hydroxyl groups [41]. These groups are commonly associated with the presence of some moisture on the surface of the samples. The heightened intensity in CA indicates a higher degree of hydration, which can facilitate the hydrolysis of both organic and inorganic components within the paint layer, accelerating its degradation. Additionally, a series of peaks between 3035 and 2810 cm^−1^ was detected in all samples, with greater intensity again in the CA sample. These peaks correspond to C-H stretching vibrations, which are associated with the paraloid introduced during conservation treatments. The strong presence of these bands in CA suggests a higher content of organic materials on the exposed layer, which can degrade over time due to photochemical reactions or environmental factors, leading to discolouration or loss of material integrity. A notable peak at 2508 cm^−1^ was identified in all samples. This feature is indicative of carbonate groups, likely originating from the calcium carbonate (CaCO_3_) [41] present in substrates. The peak at 1796 cm^−1^, present in all samples, may also relate to carbonate stretching vibrations, further supporting the presence of these compounds. The presence of a distinct peak at 1729 cm^−1^ in the CA sample, which was not observed in the other samples, is particularly notable. This peak corresponds to the carbonyl groups (C=O) in the used conservation materials (Paraloid B-72). While paraloid is effective for conservation, its degradation under UV exposure can cause yellowing or cracking, potentially contributing to the observed deterioration in the CA-related layer. Both pigment samples (CA and IS) exhibited peaks at 800, 777 and 695 cm^−1^, which can be associated with silicate minerals. A CA peak at 915 cm^−1^ was also observed, which could be related to the presence of aluminosilicates. Silicates, including various clay minerals, are often present as impurities or intentional additives in pigments [42]. The presence of these minerals might influence the pigment’s physical and chemical properties, affecting its durability. The combined evidence from these spectra helps to understand why the CA pigment experienced a more pronounced degradation processes than IS. The higher levels of moisture absorption, greater presence of degradable organic materials, and potential degradation of conservation materials like paraloid likely contribute to the poorer visual conservation state of the CA paintings.

**Figure 11:**
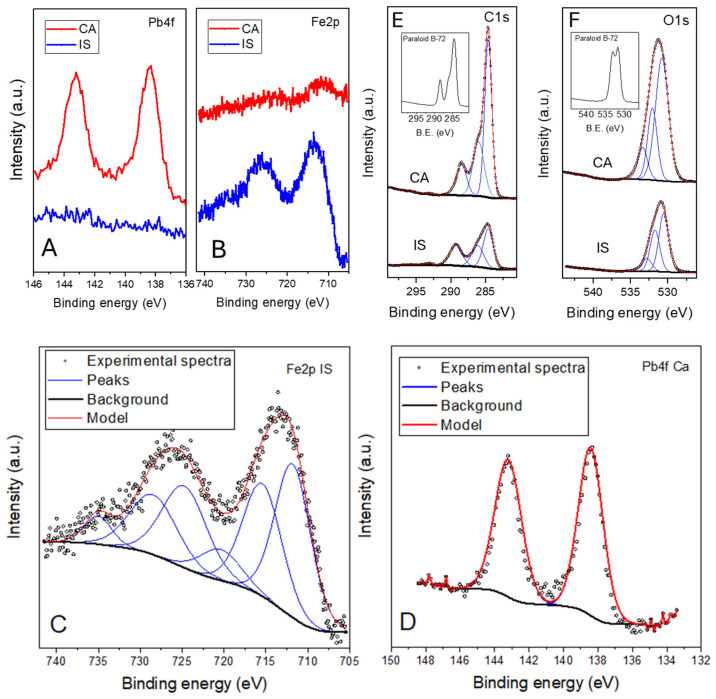
XPS results of selected samples: (**A**) spectra of Pb4f XPS region; (**B**) spectra of Fe2p XPS region; (**C**) proposed fitting of the Fe2p XPS region for the sample obtained from Isturgui; (**D**) proposed fitting of the Pb4f XPS region for the sample obtained from Cástulo; (**E**) C1s region spectra of the samples (CA and IS) and fitting, Paraloid B72 spectra (inset); and (**F**) O1s XPS region spectra of the samples (CA and IS), Paraloid B72 spectra (inset).

Figure 11A,B present the X-ray photoelectron spectra for the red pigment samples defined as CA and IS, respectively. Figure 11C shows the proposed fitting for iron (Fe2p), while Figure 11D illustrates the proposed fitting for lead (Pb4f). The XPS analysis reveals significant differences between these pigments that help explain their varying conservation statuses. The XPS spectra for sample CA identified the presence of lead at binding energies of around 137.5 eV (Pb 4f7/2) and 142.5 eV (Pb 4f5/2), and a very small amount of iron (Fe), with characteristic peaks at approximately 710.5 eV (Fe 2p3/2) and 723.0 eV (Fe 2p1/2) [43,44,45,46,47]. The detection of this element confirms the presence of lead-based compounds, such as lead red, which, as aforementioned, were widely used as Roman red pigments. Lead pigments are known for their historical use but also for their susceptibility to degradation over time [8]. Chemical reactions, such as the formation of lead sulphate or carbonate, particularly in the presence of moisture or acidic conditions, can lead to significant deterioration and are in line with the previous discussion of XRD. These reactions contribute to the observed poorer condition of sample CA, as lead-based pigments are prone to degradation, which affects both their appearance and structural integrity.

The XPS analysis of the sample obtained from Isturgi (IS) showed no detectable lead on the surface, with the binding energies for lead not observed in the spectra, but revealed a high concentration of iron-related species. The iron peaks were prominently observed at approximately 710.5 eV (Fe 2p3/2) and 723.0 eV (Fe 2p1/2), suggesting that the IS sample relies primarily on iron oxides, mainly hematite [46,47,48]. Iron oxides are known for their stability and durability, and form a more resilient pigment compared to lead-based materials. The high iron concentration contributes to the overall stability of IS, explaining its better preservation compared to CA.

In the C1s region, the CA sample shows prominent peaks at 284.6 eV (indicative of C-C or C-H bonds), 286.5 eV (C-O bonds), 288.5 eV (C=O bonds), and at around 290.0 eV (O-C=O bonds). These peaks indicate the presence of organic contaminants, surface oxidation products, and carbonates, a degradation product of lead-based compounds. In addition, as can be seen in Figure 11E (inset) these peaks are influenced by the presence of Paraloid B-72, which contains these types of carbon bonds. Conversely, the IS sample displays similar peaks but with less intensity, indicating a lower presence of these bonds and a lesser extent of degradation. In the O1s region, CA exhibits peaks at 530.0 eV (oxygen bonded to metals, such as in lead oxides), 531.5 eV (hydroxyl or carbonyl groups), and 533.0 eV (C-O bonds). In addition, the IS material shows a strong peak at 530.0 eV, indicative of stable iron oxides (Fe_2_O_3_), with less pronounced peaks at 531.5 eV and 533.0 eV, suggesting minor surface oxidation. The contrast between these pigments highlights the different degradation pathways and stability issues. Lead-based pigments like those in CA are more susceptible to environmental degradation, such as the formation of lead salts and other compounds that compromise the pigment’s integrity. This susceptibility is consistent with the poorer visual conditions observed in the CA sample. On the other hand, iron-based pigments, exemplified by IS, are generally more resistant to environmental factors, contributing to their superior preservation. Although lead was not detected on the surface of IS, it is possible that lead might be present in deeper layers or other zones of the paint not analyzed by XPS. The absence of surface lead does not exclude the possibility of lead being present elsewhere in the painting. However, the surface stability of the IS sample, coupled with its high iron content, suggests that this pigment is less susceptible to degradation compared to CA. Through XPS, other elements commonly present in samples obtained from pigments were also detected. Elements such as Ca, Si, and Al, which are often associated with the mineral matrices or the extenders used in pigment formulations, were observed. In smaller proportions, elements like S and metals such as Mg and K were detected, which might be related to the presence of impurities or secondary minerals [22]. As aforementioned, the presence of S could be particularly significant in samples containing Pb, as it might be related to sulphates.

## 4. Conclusions

Roman mural paintings are characterized by a high degree of consistency in the recipes and techniques used for their execution. However, there are slight variations in these techniques, often due to the availability of local raw materials or the choices made by the artists and owners involved in their creation. For this reason, in geographically close territories, differences in composition can be identified that directly influence their current state of conservation. The visual inspection and morphological analyses revealed a notably better conservation state for the red panels from Isturgi compared to those from Cástulo. In Cástulo’s samples, signs of advanced degradation were evident, including non-uniformly deposited layers and multiple areas of colour variation, indicating substantial structural deterioration.

Elemental analyses conducted via EDX and XRD demonstrated that the Isturgi panels primarily consist of iron oxide in the form of well-crystallized, stable hematite, while Cástulo’s panels show a combination of hematite and lead-related compounds. In addition, the XRD data for Cástulo indicate that the lead red underwent a chemical transformation that may contribute to its instability and poor conservation state. FTIR analysis further identified carbonates in both sets of samples, possibly resulting from interactions with the mortar substrate. The peaks associated with organic components are primarily linked to the use of Paraloid during the extraction process. The surface analysis conducted using XPS was consistent with FTIR and XRD results, detecting only hematite on the surface of Isturgi samples, whereas both hematite and lead-related compounds were present on those from Cástulo. The combination of these findings suggests that the less stable lead compounds, along with organic residues from previous interventions, likely contributed to the accelerated degradation observed in the Cástulo panels. Overall, these results provide valuable insights into the chemical and physical factors impacting the preservation of wall paintings, underscoring the importance of careful, informed conservation strategies in future restoration efforts.

Future research should aim for a more comprehensive characterization of Roman mural paintings by expanding the dataset to include additional archeological sites. Moreover, future research might focus on extending these techniques to other hues in the Roman palette and comparing pigment compositions across a broader geographic range within the Empire, illuminating potential trade routes and regional differences in pigment use.

A comparative study across a broader range of environmental conditions and conservation histories would provide deeper insights into the factors affecting pigment stability and deterioration. The application of advanced analytical techniques, such as synchrotron-based spectroscopy and in situ non-invasive methods, could further refine the understanding of pigment degradation mechanisms. Additionally, evaluating past conservation treatments and their long-term impact on mural stability remains a crucial area of investigation. In particular, the role of synthetic polymers like Paraloid B72 should be critically assessed to determine their influence on preservation outcomes.

Finally, the integration of digital documentation techniques, including hyperspectral imaging and 3D reconstructions, could significantly enhance the assessment and long-term monitoring of these paintings. The adoption of these technologies would contribute to the development of more precise and sustainable conservation strategies.

## Data Availability

The original contributions presented in the study are included in the article, further inquiries can be directed to the corresponding authors.
